# Antimicrobial stewardship programs in Emilia-Romagna, Italy

**DOI:** 10.1186/1753-6561-5-S6-P143

**Published:** 2011-06-29

**Authors:** A Pan, C Gagliotti, M Arlotti, P Bassi, L Bertozzi, M Borsari, C Cancellieri, R Carletti, S Giordani, M Libanore, G Magnani, P Marchegiano, E Mazzini, S Mezzadri, M Minghetti, S Nola, C Puggioli, P Ragni, G Ratti, M Sisti, C Vandelli, P Viale, P Vitali, ML Moro

**Affiliations:** 1 Area Rischio Infettivo, Agenzia Sanitari e Sociale Dell’Emilia-Romagna, Bologna, Italy; 2 Regione Emilia-Romagna, Bologna, Italy

## Introduction / objectives

To evaluate the state-of-the-art of antimicrobial stewardship programs in Emilia-Romagna we sent a questionnaire to university hospitals (UH) and Local Health Authorities hospitals (LHAH) of Emilia-Romagna.

## Methods

A multiple-choice questionnaire was sent to all public UH/LHAH of the region. The survey was constituted by 18 different questions, in 7 sections. An 8 parameters antimicrobial stewardship (AMS) score was calculated (score 0-14).

## Results

All 17 UH/LHAH completed the survey. An antimicrobial stewardship group was present in 11/17 (58%) UH/LHAH. All UH/LHAH had implemented some antimicrobial control strategies. We analysed 4 areas. A) Restricted formulary: all UH/LHAH had restricted formularies, with a median of 12 antimicrobials. B) Education: courses on surgical prophylaxis had been performed in 56% of surgical specialties, courses on antimicrobial therapy in 47% of UH/LHAH over the last year. C) Guidelines: guidelines on surgical antibiotic prophylaxis and on antimicrobial therapy were available in 100% and 71% of UH/LHAH, respectively. D) Data feed back: data on antibiotic consumption and on antimicrobial resistance were periodically fed back to the wards by 100% and 88% of UH/LHAH, respectively. The AMS score varied significantly among UH/LHAH, from 2 to 13 points.

## Conclusion

All UH/LHAH have implemented some kind of antimicrobial stewardship program, although significant differences exists between centres. To face these differences a regional project has been implemented.

## Disclosure of interest

None declared.

